# Immune insights and toxicity testing: an interview with Jeff Yoder

**DOI:** 10.1242/dmm.052639

**Published:** 2025-11-21

**Authors:** Jeff Yoder

**Affiliations:** ^1^ Genetics and Genomics Academy, North Carolina State University, Raleigh, NC 27695, USA; ^2^ Department of Biological Sciences, North Carolina State University, Raleigh, NC 27695, USA


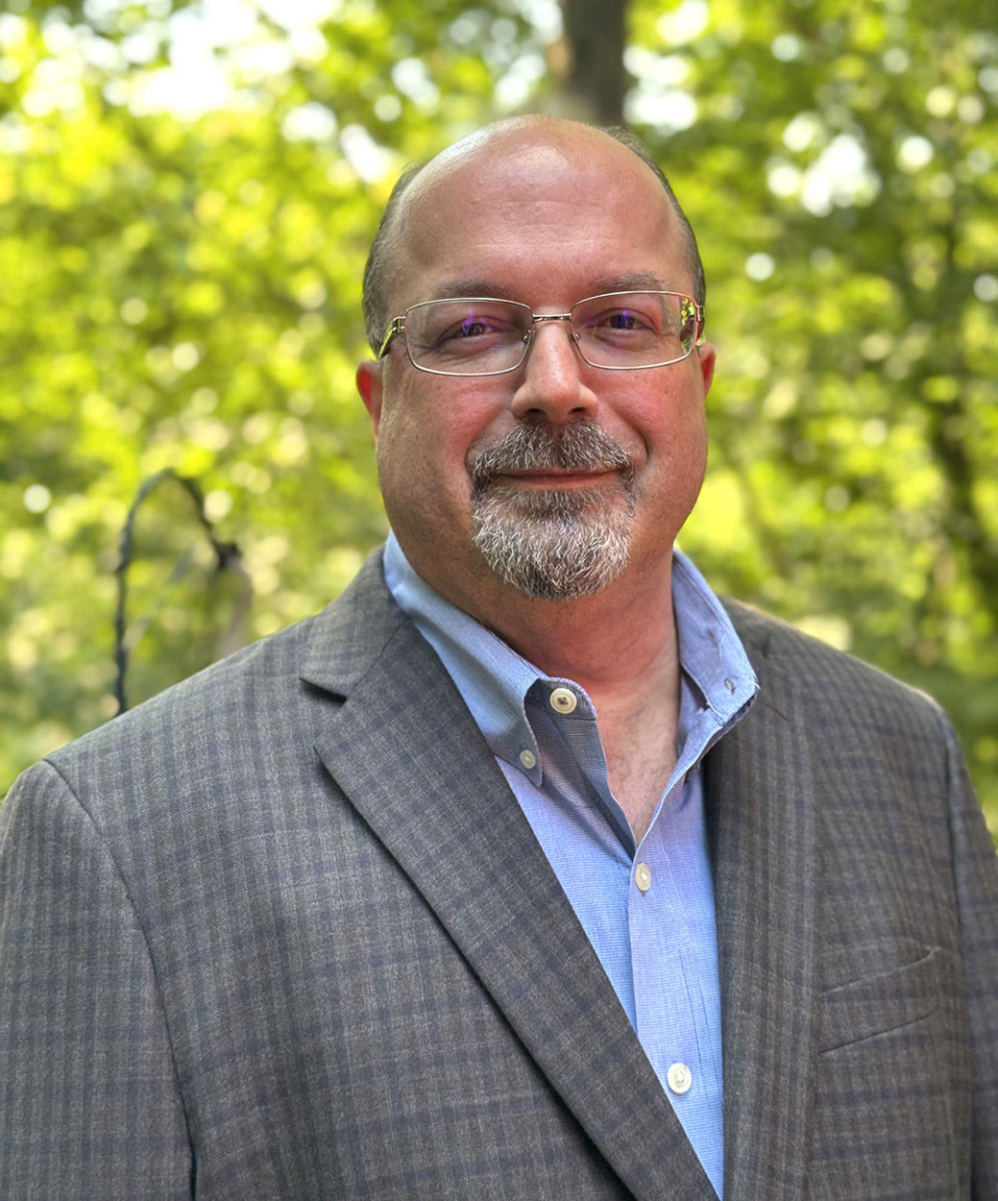


**Jeff Yoder**

Jeff Yoder is a professor at North Carolina State University, USA, where he leads a research programme in comparative immunology. His lab employs zebrafish and cell culture models, along with transcriptomic, genomic, phylogenetic and biochemical strategies, to study the molecular and functional evolution of innate immune receptors across all vertebrate lineages. In 2014, he began developing the zebrafish as a model for immunotoxicology research and currently studies how exposure to environmental contaminants adversely impacts immune function. This includes the effects of some per- and poly-fluoroalkyl substances (PFAS), often referred to as ‘forever chemicals’, that are a source of increasing concern for human health.

Jeff received his PhD in cell and developmental biology from Harvard University, USA, in 1998, working with Dr Tim Bestor on mammalian DNA methylation. As a postdoctoral fellow in Dr Gary Litman's lab at the University of South Florida, USA, Jeff shifted his research focus to comparative immunology, studying a complex family of genes encoding putative natural killer cell receptors in zebrafish. He became a faculty member at the University of South Florida before moving to North Carolina State University in 2004, where he has run a National Science Foundation- and National Institutes of Health-funded research programme for over 20 years. In 2024, Jeff was appointed Executive Director of the Genetics and Genomics Academy at North Carolina State University. The Academy works across the university to build and strengthen interdisciplinary research programmes, advance graduate training, expand genetics and genomics knowledge for all undergraduate students, and enhance state-wide outreach programmes. In this interview, we discuss fish immune systems, environmental toxicology and the impact that PFAS can have on the immune system.

**What made you decide to pursue a career in research?**

So, this goes back to high school and college for me. In high school I did well in my math and science classes but was also involved in theatre productions, both on stage and backstage, and making goofy videos with my friends at the local community television station (we volunteered to run the camera at school board meetings so we could sign out the equipment to make our videos). I honestly had a minute when I pondered the pros and cons of going to college for science or moving to Hollywood to become the next sitcom or movie star. As you might have guessed, I chose to go to college and majored in the brand-new field of biotechnology at Worcester Polytechnic Institute (WPI) in Massachusetts, USA. While in college, I didn't really have a solid plan of what would come next. I assumed that I would get a job as a lab technician. Thankfully, WPI has a significant research requirement to graduate, and I found a biophysics lab at the Worcester Foundation for Experimental Biology – which no longer exists – that would take me on for a summer research experience using fluorescence resonance energy transfer to measure the length of complementary oligonucleotides via melting curves. Since this lab was in driving distance of WPI, I convinced the lab to allow me to complete my senior thesis with them. I ended up working on cell membrane dynamics during fertilization. So, this is where I actually answer your question. A postdoc in this lab suggested that if I enjoyed research I should apply to graduate school. I initially thought there was no way I could afford to go to graduate school, but he reassured me that, in the biomedical sciences, many schools will pay you a stipend. This was amazing news to me, and I ended up applying to, I think, 11 different PhD programmes. I ended up at the Biological and Biomedical Sciences programme at Harvard Medical School with a degree in cell and developmental biology. It was really in graduate school when I realized how much I enjoyed the scientific process and discovery and that I wanted to keep doing research as long as I could get paid to do it!

**Your lab uses zebrafish both to research the immune system and in toxicology studies. What makes zebrafish a good model to address questions in these areas?**

Zebrafish larvae are a strong vertebrate model for developmental toxicology because fertilization is external, providing researchers with the ability to observe embryonic development starting from the one-cell stage. This is complemented by their rapid development that results in a beating heart and circulation within 24 h. Then, you add in that the fish are transparent for several days, permitting *in vivo* observation of organ development. Finally, the larvae are small enough that you can place them in 96-well plates for imaging and functional or behavioural studies. These features, plus the fact that you can simply expose the zebrafish to chemicals by adding them into the water, really make zebrafish a superior whole-animal model for toxicology studies.

Zebrafish larvae are also a strong model for studying innate immunity – specifically, neutrophil and macrophage function and behaviour. These haematopoietic cell lineages are fully functional in 3-day-old zebrafish larvae, and transgenic reporter lines have been developed that allow fluorescently tagged immune cells to be visualized *in vivo* in real time. Plus, I like the fact that the cells of the adaptive immune system (B and T cells) are not fully functional until the zebrafish are a few weeks old, giving us a developmental window in which we can study how the cells of the innate immune system function without the complexity of the adaptive immune system.

**What can we learn about the immune system from studying immune evolution in fish?**

We can learn a lot by studying immune gene evolution in fish. As jawed vertebrates, fish possess both an innate and an adaptive immune response and can be very useful for studying the basics of immune cell biology as well as the functional role of conserved immune genes. There are approximately 30,000 fish species on earth, which is about half of all vertebrates. Ray finned fish (Actinopterygii), which include over 96% of those 30,000 species (including zebrafish, catfish, and most of the non-shark and non-ray species), originated over 400 million years ago and, in that time, have come to occupy so many environmental niches that differ dramatically in salinity, temperature, oxygen levels and hydrostatic pressure. There are even fish that can live outside of water for extended periods of time, like lungfish and mudskippers. Thus, you can imagine the differences in the potential pathogens present in these different conditions. A lot of great work is going into studying how the genes of the immune system co-evolve with pathogen exposure, and one major thing that we've learned from studying the immune systems of fish is that there is such a wide range of strategies for fighting off pathogens. Fish encode far more Toll-like receptors than mammals, and we're just starting to identify their ligands. In addition, some fish lineages have lost significant parts of the immune system that we used to think were essential for vertebrate survival (Boehm, 2025). For example, Atlantic cod has lost MHC Class II genes but expanded their MHC Class I gene repertoire (Bjørnestad et al., 2024), while the sexual parasitism of some anglerfish species is correlated with the loss of a functional adaptive immune system (Swann et al., 2020). These are great examples to use when teaching comparative immunology. What we observe in humans and mice only represents the immune systems of 0.00003% of all vertebrates on the planet – the immunogenetic rules in humans and mice do not necessarily apply to other species. What this means is, if you want to use a fish to study human immunology, it is best to understand not only how their immune systems are similar, but also how their immune systems are different.

…if you want to use a fish to study human immunology, it is best to understand not only how their immune systems are similar, but also how their immune systems are different

**Having established your lab in the field of comparative immunology, what encouraged you to work on environmental toxicology?**

I've been running a research program at North Carolina State University, or NC State, for over 20 years now, and my interest in immunotoxicology goes back to about 10 years ago. At that time, my research program was focused on identifying novel molecular mediators of innate immunity using the zebrafish model. Multiple research groups were developing new ways to use zebrafish larvae as a whole-animal model for innate immunity, studying aspects like chemotaxis and phagocytosis. My group was moving in that direction with antisense agents to knock down gene expression (this was before CRISPR). While I was doing my comparative immunology research, there was also a long-running program in environmental toxicology ongoing at NC State. And it just so happened that the toxicology faculty had invited leadership from the US National Institute of Environmental Health Sciences (NIEHS) and Environmental Protection Agency (EPA) to campus for a meet and greet. They were looking for synergies between the research at the university and the goals of the federal agencies – NC State is less than 20 miles from research labs for the EPA and NIEHS. Somehow, I was invited to attend, and I recall introducing myself by stating “I'm not a toxicologist, but an immunologist” and explaining that the functional assays we were developing for genetic screens using zebrafish larvae could be applied to chemical screens. Dr Dori Germolec, who was the Leader of the Systems Toxicology Group at NIEHS, had an interest in immunotoxicology, and this led to funding from the NIEHS to develop the zebrafish larvae as a model for environmental immunotoxicology (Phelps et al., 2020). My first graduate student who was fully invested in using zebrafish for immunotoxicology, Drake Phelps, was very interested in working on PFAS, so he really steered us in that direction.

**Please could you give a brief explanation of what PFAS are and why they pose a threat to human and environmental health?**

So PFAS is the abbreviation for per- and poly-fluoroalkyl substances, which are a large group of man-made and highly stable compounds, some of which have been around since the 1940s. PFAS possess very useful properties like resistance to heat, water and grease, and can be found in a range of consumer products and industrial applications, including non-stick cookware, stain-resistant furniture, waterproof clothing, fast food wrappers and firefighting foam (Glüge et al., 2020). The carbon-fluorine bonds in PFAS are highly stable and contribute to their heat resistance, which is important in products like cookware, but means that they are very persistent in the environment. PFAS are introduced into waterways, land and air through industrial waste and landfills and seem to be ubiquitous across the world. Further, many PFAS have been detected in the blood of people and animals all over the world and in certain food products we eat. Unfortunately, human exposures to certain PFAS have been linked to several adverse health outcomes, including increased risks for certain cancers, decreased immune function, and the disruption of body weight regulation and metabolism (DeWitt et al, 2025; Jass et al., 2025). As one might expect, as specific PFAS are identified as being harmful to humans and the environment, new replacement PFAS are being developed and synthesized, resulting in thousands of man-made PFAS on the planet.

**What have been the most important findings from your work on the impact of PFAS on the immune system?**

I think the finding that exposure to two specific PFAS, ammonium perfluoro(2-methyl-3-oxahexanoate) (GenX) and perfluorohexanoic acid (PFHxA), suppressed the respiratory burst (a key functional immune response) using both zebrafish larvae and a human neutrophil-like cell line has been the most important finding from my group so far (Phelps et al., 2023). We screened nine different PFAS that we knew were in the rivers of North Carolina and in North Carolina residents. I feel that there are two very important impacts from this study. First, we observed that the respiratory burst assay yielded basically the same result using zebrafish larvae and human cells, demonstrating that the zebrafish larvae can serve as a reliable model for assessing the effect of chemical exposures on this specific human immune response. Second, the results from our 4-day exposure study were so important for the people who had been exposed to these chemicals in their drinking water for decades. We are continuing these studies to investigate how these PFAS suppress immune function using omics-based approaches and have other ongoing studies examining how PFAS exposures impact other important immune functions.

**What do you think are currently the greatest challenges in understanding the effects of environmental chemicals?**

I think there are two major challenges. The first challenge is the vast number of chemicals in the environment. For example, the EPA has a database that lists nearly 15,000 different PFAS, with the majority having no toxicology data (Phelps et al., 2024; USEPA, 2025). The second challenge is remediation. This is especially true for PFAS, which, as I mentioned earlier, have extremely strong chemical bonds and have been dubbed ‘forever chemicals’. The stability of PFAS means they are persistent and accumulate in the environment, in plants and in animals – including people. It is estimated that over 99% of the people in the United States have detectable levels of PFAS in their blood. We can use filtration methods to remove PFAS from drinking water, but how do we get them out of the soil, oceans, air, animals and people?

We can use filtration methods to remove PFAS from drinking water, but how do we get them out of the soil, oceans, air, animals and people?

**What key research question in your field are you most excited to see answered in the near future?**

I've been interested in natural killer (NK) cells in fish for a long time now. Newer data from single-cell transcriptome experiments really align with older cell-based studies that indicate that ray-finned fish possess NK cells. Although NK-like cell lines have been developed from the agriculturally important channel catfish (Shen et al., 2002; Yoder, 2004), there is currently no way to isolate this immune cell population from any fish. I am excited for new strategies that will allow us to identify, isolate and study the function of NK cells from ray-finned fish *in vivo* and *in vitro* – hopefully from zebrafish. It will be very exciting to see the commonalities between mammalian and fish NK cells, as well as to define the diverse cell surface receptors that mediate NK function.

**You have mentored many scientists throughout your career. What are some of the most important things to consider when mentoring junior researchers?**

I think that science communication is the most important skill for junior researchers to develop. This includes communicating with other scientists, through research talks, research papers and grant proposals, and then communicating with the public through outreach events. Being able to give a research seminar that does not get into experimental ‘weeds’ is an important skill to develop, especially when speaking to an audience with various scientific backgrounds. And being able to give talks that tell a ‘story’ at different levels can make someone a great communicator – emphasizing to the audience “why they should care” is so important.

**If you weren't a scientist, what career do you think you would have pursued?**

As I mentioned earlier, when I was in high school and college, I was involved with a number of theatre and local television productions and used to joke that my back-up career was as a game show host.
